# Cerebral Venous Thrombosis: A Mimic of Brain Metastases in Colorectal Cancer Associated with a Better Prognosis

**DOI:** 10.1155/2013/109412

**Published:** 2013-06-13

**Authors:** Nida Iqbal, Atul Sharma

**Affiliations:** Department of Medical Oncology, Dr. B. R. A. Institute Rotary Cancer Hospital, All India Institute of Medical Sciences, New Delhi 110029, India

## Abstract

Malignancy is known to be one of the predisposing factors of cerebral venous thrombosis (CVT) due to its hypercoagulable state. CVT is a rare disorder which can lead to frequent misdiagnoses of brain metastases in such cases. We report here the case of a 35-year-old female with metastatic colon adenocarcinoma presenting with sudden neurological symptoms. Brain MRI and magnetic resonance venography confirmed the presence of CVT. She was treated with low molecular weight heparin followed by warfarin. She recovered and is doing well on warfarin after 5 months of diagnosis of CVT. CVT should be strongly suspected as a cause of neurological dysfunction in any case of disseminated malignancy including colon adenocarcinoma. Rapid diagnosis and initiation of therapy should be considered because of its favourable outcome.

## 1. Introduction

Cerebral venous thrombosis (CVT), that is, any thrombosis that occurs in intracranial veins or sinuses, [1] is a rare disorder affecting approximately 5 persons per million per year with huge regional variations [2]. It accounts for approximately 0.5% of all the strokes and most commonly affects young adults. Many disorders can either cause or just predispose to CVT. They include medical, surgical, and obstetrical causes of deep vein thrombosis, genetic and acquired prothrombotic disorders, cancer and hematological disorders, inflammatory systemic disorders, pregnancy and puerperium, infections, and local causes such as tumors, arteriovenous malformations, trauma, central nervous system infections, and infections of the ear, sinus, mouth, face, and neck [1]. Cerebral venous thrombosis has a wide spectrum of clinical manifestations and modes of onset that may mimic many other neurological disorders and lead to frequent misdiagnosis and delay in treatment. Before the advent of computed tomography (CT) and magnetic resonance imaging (MRI), CVT was usually diagnosed at autopsy. It has got a favourable outcome with case fatality of less than 10% [1]. Cerebral venous thrombosis is a rare paraneoplastic syndrome.

Herein, we report a case of metastatic colorectal cancer who developed cerebral venous thrombosis while on chemotherapy.

## 2. Case Report

**Figure 1:**
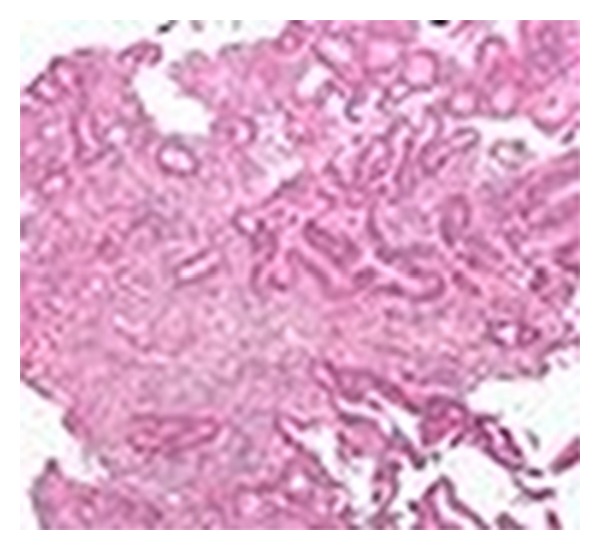
H&E of colon showing invasive adenocarcinoma.

**Figure 2:**
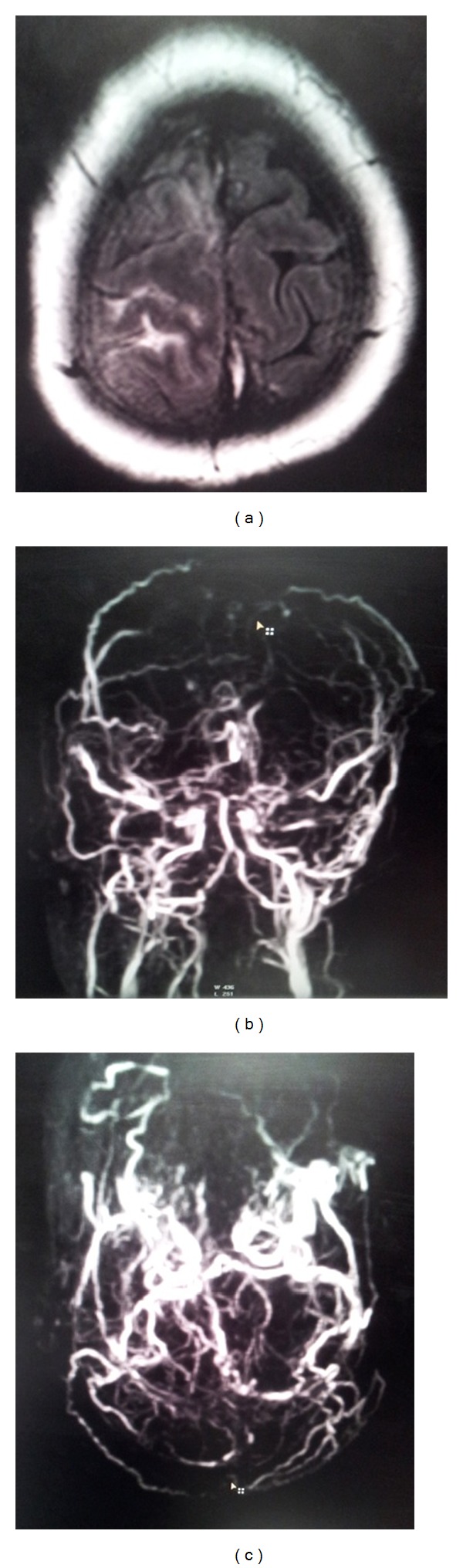
Brain MRI and magnetic resonance (MR) venography (AP view). (a) Hemorrhagic infarction was noted in the right parietal lobe on T2-weighed imaging. (b), (c) MR venography showed occlusion of the superior and inferior sagittal sinuses, both transverse sinuses, and bilateral sigmoid sinuses.

A 35-year-old female, a diagnosed case of metastatic colorectal adenocarcinoma, (Figure 1) was on palliative chemotherapy with capecitabine and irinotecan (CAPIRI) when she presented with the history of sudden onset of headache, blurring of vision, weakness of left side of body, and multiple episodes of vomitings. She had no history of hypertension, diabetes, pregnancy or use of oral contraceptives. On physical examination, she was conscious, oriented, and having mild dysarthria and grade 3 power of left side of the body. There were no signs of meningismus. Fundus examination revealed bilateral papilledema. Contrast enhanced CT scan of head revealed mild edema in right frontal lobe. Brain MRI revealed hemorrhagic infarct in right parietal lobe (Figure 2(a)), and magnetic resonance (MR) venography revealed occlusion of superior and inferior sagittal sinuses, both transverse sinuses, and bilateral sigmoid sinuses with thrombus (Figures 2(b) and 2(c)). Laboratory parameters revealed a normal complete blood count, sedimentation rate, blood sugar, lipid profile, and kidney and liver function tests. Electrocardiogram and echocardiography were also normal.

To rule out any coexisting prothrombotic condition, homocysteine levels, protein C, protein S, factor V, and antithrombin III were done which were within normal limits. For disease assessment, contrast enhanced CT scan of abdomen and pelvis was done which revealed the disease to be in complete remission. She was started on low molecular weight heparin. There was marked improvement in her symptoms, and she was discharged in stable condition on oral warfarin. She is doing well even after 5 months from the diagnosis of cerebral venous thrombosis.

## 3. Discussion

Cerebrovascular events may be the first clinical manifestations in patients with underlying malignancy or may develop subsequently during the course. Systemic thrombosis like deep vein thrombosis or pulmonary embolism is well recognized in cancer patients, although CVTs are uncommon in cancer. Potential mechanisms for an association of cancer with cerebral venous thrombosis (CVT) include direct tumor compression, tumor invasion of cerebral sinuses, the hypercoagulable state associated with cancer, or the chemotherapeutic side effects [3–5]. Cerebral venous thrombosis has been reported to be associated with various cancers like squamous cell cervical cancer [6], non-Hodgkin's lymphoma [7], and breast cancer [8]. Cerebral sinus venous thrombosis associated with chemotherapy has so far been described in a patient with colon cancer treated with FOLFIRI/bevacizumab [9], in a patient with a brain tumor treated with temozolomide, focal brain radiotherapy plus bevacizumab [10], in an adolescent with Ewing sarcoma treated with cisplatin, ifosfamide, adriamycin, and vincristine [11], and in two patients of nonseminomatous germ cell tumor treated with cisplatin, bleomycin, and etoposide [12]. Like our case, a case of cerebral venous thrombosis in a patient of rectal cancer has been described, but that was associated with cerebral metastases also [13].

Cerebral venous thrombosis should always be kept in the differential diagnosis of any form of neurological symptoms in a patient with cancer even without cerebral metastases and even if the disease is in complete remission as in our case. As CVT has got a favourable outcome unlike other neurological syndromes, early diagnosis with MRI/MR venography and rapid institution of therapy with heparin should be considered.

## References

[1] Ferro JM, Canhão P, Stam J, Bousser M-G, Barinagarrementeria F (2004). Prognosis of cerebral vein and dural sinus thrombosis: results of the international study on cerebral vein and dural sinus thrombosis (ISCVT). *Stroke*.

[2] Bousser M-G, Ferro JM (2007). Cerebral venous thrombosis: an update. *The Lancet Neurology*.

[3] Raizer JJ, DeAngelis LM (2000). Cerebral sinus thrombosis diagnosed by MRI and MR venography in cancer patients. *Neurology*.

[4] Rogers LR (2004). Cerebrovascular complications in patients with cancer. *Seminars in Neurology*.

[5] Astudillo L, Lacroix-Triki M, Cousin F, Chevreau C (2007). A rarely diagnosed paraneoplastic syndrome: cerebral venous thrombosis. *Revue de Medecine Interne*.

[6] López-Peláez MF, Millán JM, De Vergas J (2000). Fatal cerebral venous sinus thrombosis as major complication of metastatic cervical mass: computed tomography and magnetic resonance findings. *Journal of Laryngology and Otology*.

[7] Enevoldson TP, Ross Russell RW (1990). Cerebral venous thrombosis: new causes for an old syndrome?. *Quarterly Journal of Medicine*.

[8] Soda T, Edagawa K, Tsuji K, Dehara M, Nakajima Y, Ito M (2008). A case of deep cerebral venous thrombosis associated with breast cancer. *Clinical Neurology*.

[9] Ozen A, Cicin I, Sezer A (2009). Dural sinus vein thrombosis in a patient with colon cancer treated with FOLFIRI/bevacizumab. *Journal of Cancer Research and Therapeutics*.

[10] Vargo JA, Snelling BM, Ghareeb ER (2011). Dural venous sinus thrombosis in anaplastic astrocytoma following concurrent temozolomide and focal brain radiotherapy plus bevacizumab. *Journal of Neuro-Oncology*.

[11] Unal E, Yazar A, Koksal Y, Caliskan U, Paksoy Y, Kalkan E (2008). Cerebral venous sinus thrombosis in an adolescent with Ewing sarcoma. *Child’s Nervous System*.

[12] Papet C, Gutzeit A, Pless M (2011). Two cases of cerebral sinus venous thrombosis following chemotherapy for non-seminomatous germ cell tumor. *Case Reports in Oncology*.

[13] Tóth L, Szakáll S, Káposzta Z, Udvardy M (2000). Cerebral deep vein thrombosis associated with rectal cancer. *Orvosi Hetilap*.

